# The Extract of *Piper nigrum* Improves the Cognitive Impairment and Mood in Sleep-Deprived Mice Through the JAK1/STAT3 Signalling Pathway

**DOI:** 10.3390/ijms26051842

**Published:** 2025-02-21

**Authors:** Dongyan Guan, Zhiying Hou, Bei Fan, Yajuan Bai, Honghong Wu, Jiawei Yu, Hui Xie, Zhouwei Duan, Fengzhong Wang, Qiong Wang

**Affiliations:** 1 Institute of Food Science and Technology, Chinese Academy of Agricultural Sciences, Beijing 100193, China; gdy9512@163.com (D.G.); 82101231155@caas.cn (Z.H.); fanbei517@163.com (B.F.); xbaizxyq@sina.com (Y.B.); whh18322839572@163.com (H.W.); ycloverjswmart@163.com (J.Y.); 2 National Nanfan Research Institute (Sanya), Chinese Academy of Agricultural Sciences, Sanya 572024, China; 3 Institute of Processing & Design of Agroproducts, Hainan Academy of Agricultural Science, Haikou 571100, China; xiehuinky@163.com (H.X.); universeduan@163.com (Z.D.)

**Keywords:** piper extract, network pharmacology, sleep deprivation, cognitive impairment

## Abstract

*Piper nigrum* L. (PN), which contains various bioactive compounds, is a plant with homologous medicine and food. Sleep deprivation (SD) profoundly impacts cognitive function and emotional health. However, the mechanisms by which PN improves cognitive function and depressive mood induced by SD remain unclear. In our study, network pharmacology and molecular docking techniques were used to predict the potential mechanisms by which PN regulates SD. In this study, 220 compounds were identified in PN, and 10 core targets were screened through network pharmacology. Animal experiments showed that PN ameliorated depressive mood and cognitive deficits in sleep-deprived mice, upregulated the serum activities of superoxide dismutase (SOD), glutathione (GSH), and catalase (CAT), and downregulated malondialdehyde (MDA) levels. The ELISA assay showed that PN significantly decreased the tumour necrosis factor-alpha (TNF-α), interleukin-6 (IL-6), and interleukin-1 beta (IL-1β) levels. Histopathological staining of brain tissue demonstrated that PN mitigates SD-induced hippocampal damage, enables the hippocampus to produce more neurotransmitters, including 5-hydroxytryptamine (5-HT), gamma-aminobutyric acid (GABA), and dopamine (DA), and reduces glutamate (Glu) levels. RT-qPCR and WB analyses further indicated that PN could exert anti-SD effects by inhibiting the over-activation of the JAK1/STAT3 signalling pathway. In the PC12 cell model, PN could reduce inflammation and prevent apoptosis, exerting neuroprotective effects. In summary, PN has positive effects on alleviating depressive symptoms and cognitive dysfunction induced by SD.

## 1. Introduction

Sleep is an essential component of a daily routine, serving as a critical period for repair and recovery, thus sustaining human health. However, due to global modernisation, high work-related stress, and enhanced screen time owing to dependency on electronic devices, sleep time is significantly reduced. Sleep deprivation (SD) has emerged as a serious norm in modern life. In the USA, about 32% of working adults get less than six hours of sleep per day [1]. In addition, sleep time among children and teenagers is also gradually declining [2]. Gender plays a crucial role in the cognitive effects of SD. Numerous studies have shown that cognitive performance following SD differs significantly between sexes [3,4]. Chronic lack of sleep adversely affects both physical and mental health and appears to be one of the major contributing factors to memory decline, cognitive dysfunction, and mood disorders. These elements are significant contributors to mental health conditions, including depression [5]. Therefore, it is necessary to clearly understand the effects of SD on cognitive impairment and mood disorders.

SD is closely associated with oxidative stress, inflammatory responses, and disruptions in neurotransmitter metabolism [6]. Recent studies highlighted the elevated oxidative stress in the brains of sleep-deprived individuals [7,8]. SD could further trigger the inflammatory response in the body, leading to inflammatory dysregulation [9]. The excessive expression of inflammatory cytokines further damages hippocampal neurons and synaptic plasticity. Furthermore, SD disrupts the metabolic balance of monoamine neurotransmitters, reducing the levels of key neurotransmitters, such as dopamine (DA), serotonin (5-HT), and γ-aminobutyric acid (GABA), thereby impairing learning, memory, and emotional regulation [10,11]. These factors interact to exacerbate the damage that SD causes to the central nervous system.

*Piper nigrum* L. (PN) is a climbing vine of the genus *Piper* in the family Piperaceae, renowned for its significant culinary and medicinal value. The fruit of PN contains various bioactive compounds, with piperine being the primary active ingredient [12]. Studies have shown that piperine can enhance the bioavailability of certain drugs and exert regulatory effects on the nervous and immune systems [13]. In addition, the fruit of PN contains volatile oil components, such as terpenes and aromatic compounds, which primarily contribute to its characteristic aroma [14]. It has been shown that certain polyunsaturated fatty acids in PN, such as oleic acid and linoleic acid, offer significant cardiovascular health benefits [15]. Previous studies demonstrated that PN has anti-inflammatory effects and could be beneficial in depression and sleep improvement [16,17]. However, the mechanism of this action is unclear.

Network pharmacology is an emerging approach in drug research. Unlike traditional drug discovery methods that typically focus on a single target or mechanism, network pharmacology reveals the multi-layered and multi-target mechanisms of drug action by systematically analysing the complex relationships between drugs, targets, diseases, and biomolecules [6]. The use of network pharmacology techniques prompted us to identify the complex interactions between various components of traditional Chinese medicine (TCM) and multiple targets, predicting potential pharmacological pathways [18]. This approach may provide a systematic comprehension of the mechanism of TCM, facilitating the scientific advancement of TCM development. Currently, there are few studies on PN in the treatment of SD.

In this study, to explore the mechanisms underlying the action of PN in ameliorating SD, we first identified the major bioactive compounds in PN using LC-MS/MS (*liquid chromatography-mass spectrometry/mass spectrometry*). Subsequently, network pharmacology and molecular docking techniques were used to predict 10 potential targets and key pathways through which PN may regulate SD. Finally, dual validation was validated using an in vivo SD mouse model and in vitro PC12 cell lines. The results suggest that PN can alleviate inflammation and oxidative stress in SD mice and may improve the cognitive and emotional disturbances induced by SD by inhibiting excessive activation of the JAK1/STAT3 signalling pathway. Our findings will contribute to the development of PN applications for functional and healthy foods.

## 2. Results

### 2.1. Chemical Component Identification in PN

A total of 220 active components were found in the PN using LC-MS/MS. The primary ingredients residing in PN are presented in Table 1. The total ion chromatogram is depicted in Figure S1A,B. The content of piperine in the PN was predominant and constituted around 64.67%, as determined using high-performance liquid chromatography (*HPLC*) (Figure S1C).

### 2.2. Active Ingredient and Target Collection in PN

**Figure 1:**
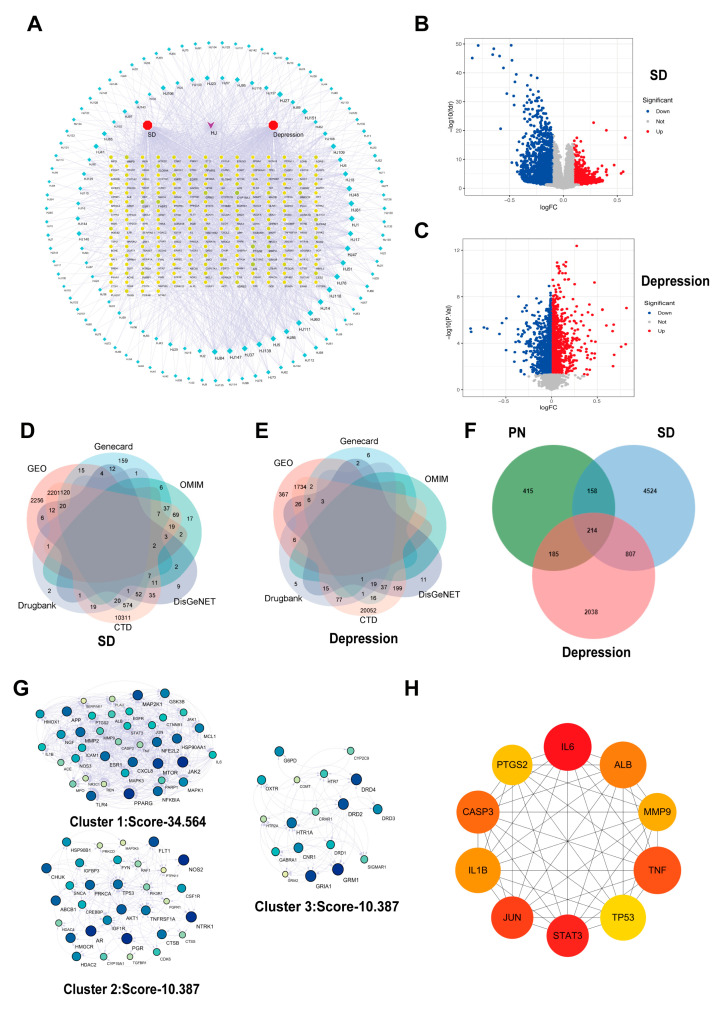
Collection of active ingredients and disease targets in PN. (**A**) Network of targets with PN active components. Green diamonds represent components, and yellow circles represent targets. (**B**) Volcano map of SD-related differential genes screened from the GSE208668 dataset; (**C**) Volcano map of differential genes associated with depression screened from the GSE98793 dataset. (**D**) Collection of SD-related targets from five disease databases and the GSE208668 dataset; (**E**) Collection of depression-related targets from five disease databases and the GSE98793 dataset. (**F**) Venn diagram of targets shared by SD, depression, and the active components of PN. (**G**) Three protein clusters were identified through MCODE plugin analysis. (**H**) The top 10 core genes were obtained from analysis using the cytoHubba plugin.

The 220 compounds identified via LC-MS/MS were queried using PubChem. A total of 972 targets were identified from 154 active components after eliminating duplicate targets. A network of the active ingredients and their targets in PN was constructed using Cytoscape 3.8.2 (Figure 1A). Additionally, three key actives in piperine were screened based on degree values: piperine, dibutyl phthalate, and pentaporphyrinl.

### 2.3. Disease Target Collection and Analysis

Gene expression profiles related to SD were searched in the GEO database, selecting GSE208668 as the study sample. As presented in Figure 1B, 970 upregulated genes and 3698 downregulated genes associated with SD were screened (*p* < 0.05, |logFC| > 1). Similarly, GSE98793 was selected as a study sample for depression, and 1347 upregulated genes and 1215 downregulated genes (Figure 1C) were identified. Furthermore, 8317 target genes associated with SD in the Gene Card database were identified, of which 1037 potential targets had a relevance score of ≥10. In addition, for the treatment of SD, 182, 45, 177, and 176 potential targets were identified from the OMIM, Drug Bank, DisGeNET, and CTD databases, respectively. The intersection of potential targets that appeared at least twice across these databases was selected (Figure 1D). Similarly, 14,838 targets screened were associated with depression from the Gene Cards database, of which 93 potential targets had a relevance score ≥ 10. Additionally, 1, 127, 319, and 22,179 potential depression treatment targets were obtained from the OMIM, Drug Bank, DisGeNET, and CTD databases, respectively, and those that appeared at least twice were screened out (Figure 1E).

By intersecting the predicted targets of PN with SD and depression disease targets, 214 common targets were identified (Figure 1F). To better understand the functions of these targets, a clustering analysis was performed using the MCODE plugin, revealing three distinct protein clusters (Figure 1G), with the highest scoring node module consisting of 39 nodes and 635 edges (Cluster 1: Score-34.564). Using the cytoHubba plugin, ten core targets were identified among these differential genes: IL6, STAT3, JUN, TNF, CASP3 (Caspase3), ALB, IL-1β, MMP9, TP53, and PTGS2 (Figure 1H). This suggests their potential critical regulatory roles in the protein interaction network.

### 2.4. GO and KEGG Enrichment Analysis

**Figure 2:**
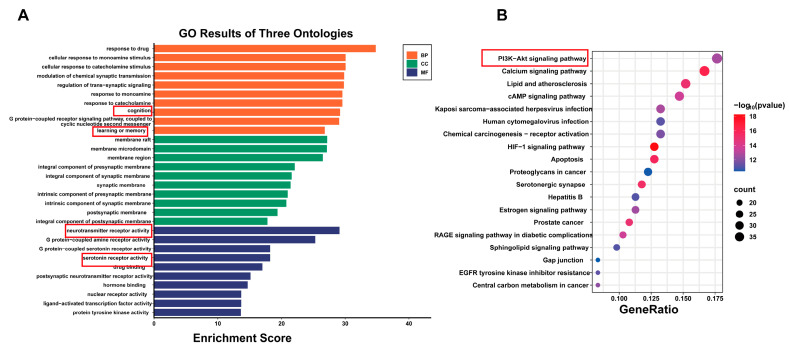
Analysis of KEGG and GO enrichment analysis. (**A**) GO results of three ontologies. (**B**) Top 20 pathway enrichment analysis.

To further understand the mechanisms by which PN improves SD, the obtained key targets were analysed for GO functional enrichment, including analyses of biological processes (BP), cellular components (CC), and molecular functions (MF). It was revealed that BP primarily involves cognition, learning or memory, and modulation of trans-synaptic signalling. CC mainly pertains to membrane rafts, membrane microdomains, and synaptic membranes, while MF is predominantly associated with neurotransmitter receptor activity and serotonin receptor activity (Figure 2A). Additionally, the KEGG pathways were mainly enriched in the PI3K-AKT and serotonergic synapse signalling pathways (Figure 2B). Each pathway interacts with common targets, implying that PN may improve cognitive impairment and depressive symptoms induced by SD by coordinating multiple pathways.

### 2.5. Molecular Docking Results

**Figure 3:**
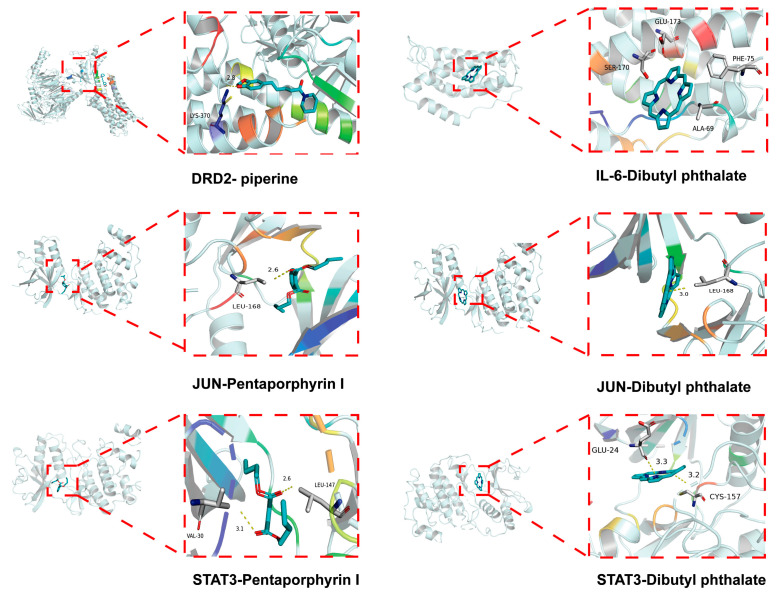
Molecular docking results.

To further validate the network pharmacology results obtained above, the molecular docking of the core targets dopamine receptor D2 (DRD2), IL-6, JUN, and STAT3 with the main active compounds piperine, dibutyl phthalate, and pentaporphyrinl was conducted. The results of these compounds exhibited binding energies ranging from −9.5 to −5.3 kcal/mol with their respective target proteins, demonstrating appreciable binding affinities (Figure 3).

### 2.6. PN Improves Behaviour in SD Mice

**Figure 4:**
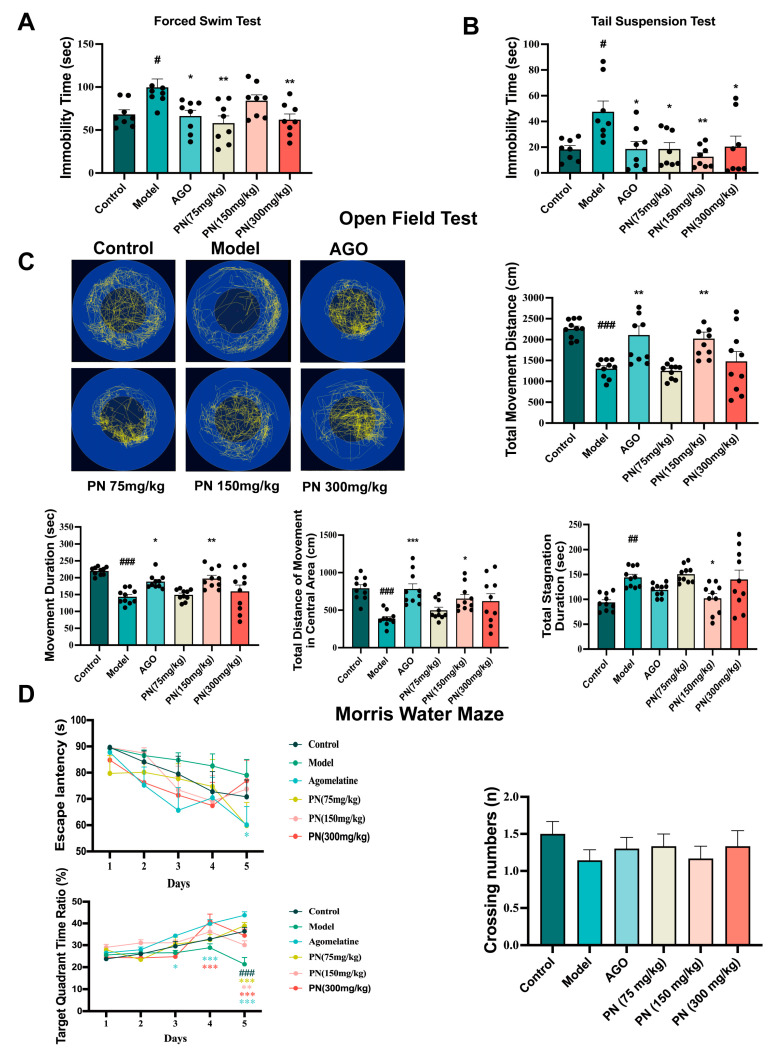
Effects of PN on the behaviour of SD mice. (**A**) FST in SD mice; (**B**) TST in SD mice; (**C**) OFT in SD mice; (**D**) MWM in SD mice. # *p* < 0.05, ## *p* < 0.01, ### *p* < 0.001 compared to the control. * *p* < 0.05, ** *p* < 0.01, *** *p* < 0.001 compared to the model group (*n* = 8–10, mean ± SEM).

The behavioural performance of SD mice was assessed using FST, TST, and OFT. As shown in Figure 4A,B, the SD model group had a longer resting time than the control group. In contrast, the AGO and PN (75 and 300 mg/kg) groups dramatically reduced the immobility period in the SD mice in the FST and TST. PN (150 mg/kg) only improved the immobility time of the mice in the TST. In the OFT, AGO and PN (150 mg/kg) significantly increased the total distance travelled, the central area distance, and the active time and decreased the stagnation time (Figure 4C). The efficacy of PN was also evaluated for its ameliorating potential of cognitive decline among SD mice using MWM (Figure 4D). In the MWM test, the escape latency on day 5 was significantly higher, with reduced quadrant area time spent by SD mice compared to control mice. On days 3 and 4, the residence time in the target quadrant of the mice in the AGO and PN (150 mg/kg) groups increased dramatically. Similarly, on day 5, the residence time of mice in the target quadrant was significantly higher in all treatment groups than in the model group. However, PN treatment had no notable effect on crossing numbers. These behavioural tests suggest that PN probably ameliorates SD-induced depression and cognitive deficits.

### 2.7. PN Alleviates Oxidative Stress and Inflammatory Levels in SD Mice

**Figure 5:**
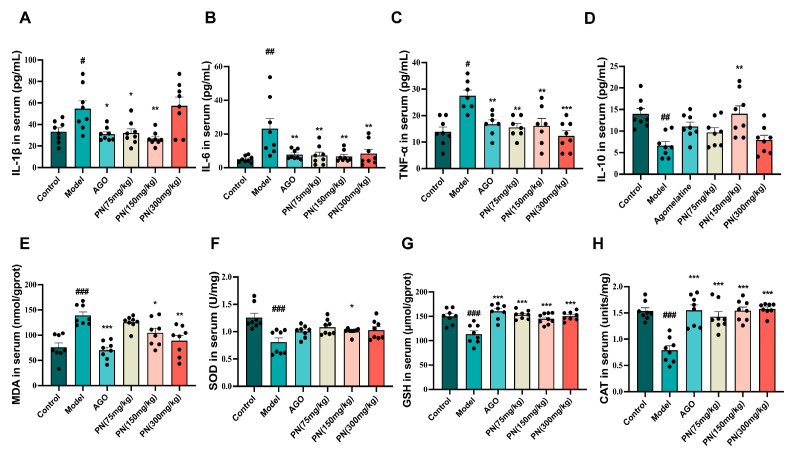
Effect of PN on the biochemical index in SD mice. (**A**–**D**) Inflammatory factor levels in SD mice; (**E**–**H**) Oxidative stress levels in SD mice. # *p* < 0.05, ## *p* < 0.01, ### *p* < 0.001 compared to the control. * *p* < 0.05, ** *p* < 0.01, *** *p* < 0.001 compared to the model group (*n* = 6–8, mean ± SEM).

According to network pharmacology results, IL-6, IL-1β, TNF-α, and IL-10 are closely associated with SD and depression. Therefore, the levels of four inflammatory markers were assessed (Figure 5A–D). The SD mice showed drastically higher levels of three inflammatory markers (IL-6, IL-1β, and TNF-α) compared to the control mice, while the level of IL-10 was significantly decreased. The levels of all these inflammatory factors in the AGO and PN treatment groups were considerably lower than in the model group. PN (150 mg/kg) treatment could reverse the IL-10 levels. There is a mutually reinforcing relationship between oxidative stress and an inflammatory response. It appeared that SD may induce oxidative stress in mice (Figure 5E–H). Following PN and AGO treatments, the levels of GSH and CAT were significantly higher, while MDA levels were noticeably lower than those in the model group. PN treatment had a negligible effect on SOD levels, with only PN at 150 mg/kg showing a decline.

### 2.8. PN Improves Hippocampal Neuronal Cell Damage in SD Mice

**Figure 6:**
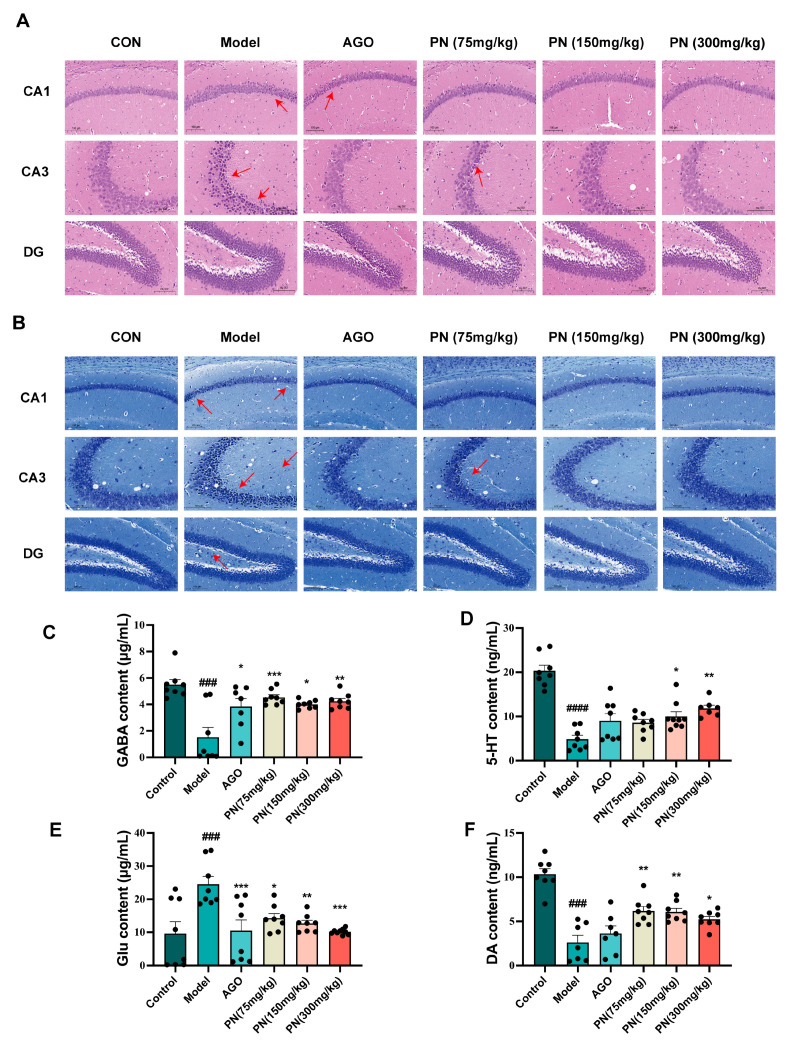
PN improves hippocampal neuronal cell damage in SD mice. (**A**) HE staining of the hippocampus in SD mice; (**B**) Hippocampal Nissl staining in SD mice. The scale of hippocampal tissue is 100 μm; red arrows indicate areas of injury. (**C**–**F**) Results of neurotransmitter measurements in the hippocampus. ### *p* < 0.001, #### *p* < 0.0001 compared to the control. * *p* < 0.05, ** *p* < 0.01, *** *p* < 0.001 compared to the model group (*n* = 6–8, mean ± SEM).

As shown in Figure 6A, the HE staining results revealed that the hippocampal neurons in each group exhibited relatively regular morphology and clear structures. However, in the model group, neuronal cells in the CA1 and CA3 regions displayed a loose arrangement, nuclear pyknosis, and hyperchromasia, indicating significant damage. After PN and AGO treatments, the hippocampal neurons in mice gradually recovered with reduced nuclear pyknosis. Nissl staining showed that neurons in the model group exhibited nuclear pyknosis and vacuolation, indicating neuronal damage (Figure 6B). After PN and AGO treatments, neurons in the hippocampal CA3 and DG regions showed varying degrees of improvement.

### 2.9. PN Regulates Neurotransmitter Levels in SD Mice

Neurotransmitters such as GABA, 5-HT, Glu, and DA are closely associated with sleep. SD resulted in decreased levels of GABA, 5-hydroxytryptamine, and DA in the hippocampus of mice, whereas levels of Glu were significantly increased (Figure 6C–F). After PN and AGO treatments, GABA and DA levels in the hippocampus of mice could be reversed, and Glu levels could be reduced. AGO treatment had no significant effect on the 5-HT levels in the hippocampus of SD rats, whereas PN (150 and 300 mg/kg) treatment significantly reduced the 5-HT levels in the hippocampus of SD rats (Figure 6D).

### 2.10. RT-qPCR Validation of Key Gene Expression Levels

**Figure 7:**
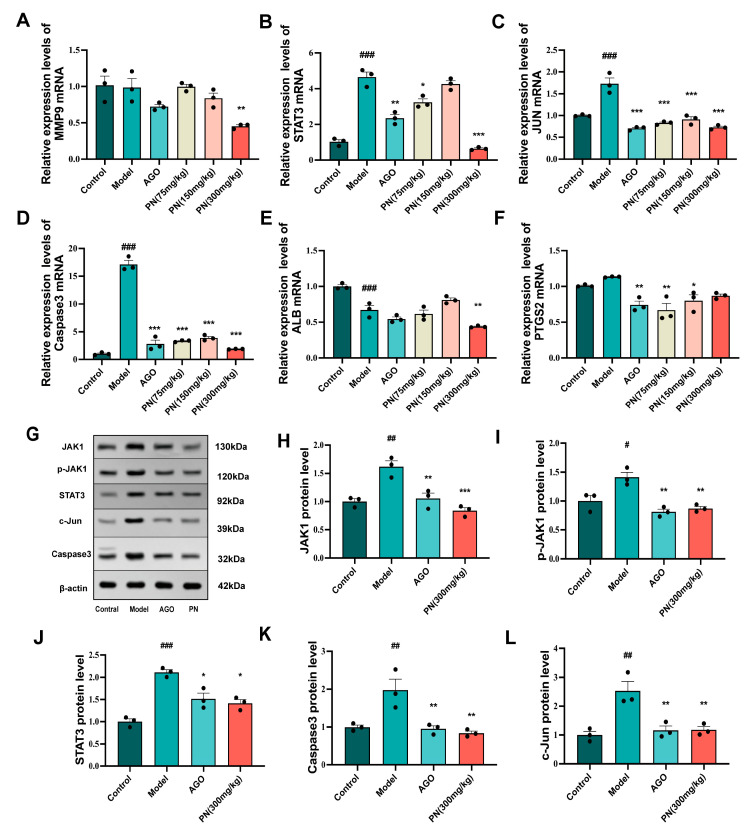
Effects of PN on the expression of core targets in the hippocampal tissue of SD mice. (**A**–**F**) Results of the key gene mRNA expression; (**G**–**L**) Results of the key protein expression assays. # *p* < 0.05, ## *p* < 0.01, ### *p* < 0.001 compared to the control. * *p* < 0.05, ** *p* < 0.01, *** *p* < 0.001 compared to the model group (*n* = 3, mean ± SEM).

The expression levels of key target genes identified by the network pharmacology screen were assessed by RT-qPCR to validate predictions. The mRNA levels of STAT3, JUN, and Caspase3 were drastically elevated in the hippocampus of the model group mice, while reduced mRNA levels of ALB were noticeable (Figure 7B–E). The JUN and Caspase3 mRNA levels were markedly downregulated in the PN and AGO treatment groups, while PTGS2 and STAT3 mRNA levels were similarly decreased after AGO and PN (75 mg/kg or 300 mg/kg) treatments. However, only the PN (300 mg/kg) treatment group exhibited a reduction in MMP9 and ALB mRNA levels, suggesting a modest effect (Figure 7A,E).

### 2.11. Effect of PN on Protein Expression in SD Mice Hippocampal Tissue

Further WB analysis revealed that SD-induced hippocampal damage in mice significantly upregulated the expression of JAK1, p-JAK1, and STAT3 proteins, as well as markedly increasing the levels of pro-apoptotic proteins Caspase3 and c-Jun (Figure 7H–L). PN and AGO treatments effectively suppressed the expression of JAK1 and STAT3 proteins in the hippocampus of SD mice and, by modulating the JAK1/STAT signalling pathway, further inhibited the expression of c-Jun and Caspase3 proteins.

### 2.12. Protective Effect of PN on PC12 Cells

**Figure 8:**
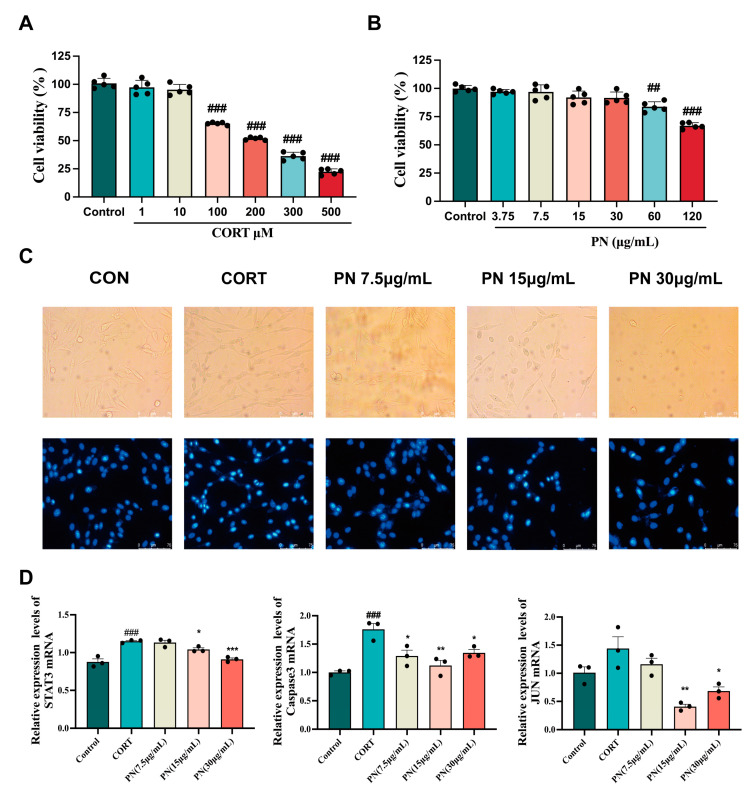
Protective effect of PN on PC12 cells. (**A**) Impact of various CORT concentrations on PC12 cell survival; (**B**) Effect of different concentrations of PN on PC12 cell viability; (**C**) Fluorescence microscopic observation of the effect of PN on PC12 cell apoptosis; (**D**) Effects of PN on the expression of key genes in the PC12 cell. ## *p* <0.01, ### *p* < 0.001 compared to the control. * *p* < 0.05, ** *p* < 0.01, *** *p* < 0.001 compared to the model group (*n* = 3, mean ± SEM).

As shown in Figure 8A, CORT decreased PC12 cell viability in a concentration-dependent manner, with the optimal induction concentration being 200 μM. Similarly, PN had no toxicity to PC12 cells at concentrations below 60 μg/mL. Therefore, PN (7.5, 15, and 30 μg/mL) concentrations were selected for subsequent studies (Figure 8B). Furthermore, Hoechst 33,258 staining revealed that the nuclei of PC12 cells treated with CORT (200 μM) appeared fragmented and intensely stained, exhibiting a whitish colour under a fluorescence microscope (Figure 8C). However, treatment with PN (7.5, 15, and 30 μg/mL) effectively reversed the apoptosis. RT-qPCR results exhibited that PN (15 and 30 μg/mL) significantly reduced STAT3, Caspase3, and JUN mRNA expression in PC12 cells (Figure 8D), thereby supporting the results obtained from animal experiments.

## 3. Discussion

Indeed, SD is increasingly prevalent in modern society. Ample research indicates that prolonged SD possibly leads to cognitive impairments manifested as decreased attention and memory deficits. Currently, the primary treatment for SD and associated neuropsychiatric conditions, such as depression, anxiety, and memory impairment, involves the use of sedative hypnotics and antidepressants. However, the side effects associated with these medications are becoming increasingly evident [19,20]. TCM can act on the body through multiple components and targets, with fewer side effects and a lower risk of dependency and addiction, such as Xiaoyao San [21,22].

PN is a spice with both food and medicinal applications. In recent years, its pharmacological effects have received increasing attention [23,24]. In our study, three major active compounds residing in PN (piperine, dibutyl phthalate, and pentaporphyrinl) were predicted by network pharmacology and screened for 10 core targets. Behavioural experiments demonstrated that the intervention of PN alleviated depression-like behaviour in SD mice. PN essential oil has been shown to improve the depressive behaviour of mice in the TST and OFT experiments and to exert a dual effect of anxiolytic and antidepressant properties by engaging with the serotonergic transmission system [25]. Black PN ethanol extract significantly reduces immobility time in mice during the TST and OFT, demonstrating notable antidepressant and anxiolytic effects [26]. In addition, studies have revealed that SD affects learning and memory abilities in mice [27]. Piperine likely inhibits the activity of acetylcholinesterase (AChE), thereby increasing acetylcholine levels and subsequently enhancing memory [28]. This may be one of the pathways through which PN improves cognitive function in mice, which is consistent with our findings.

An increasing number of studies indicate that SD exacerbates oxidative damage in the brain [29,30]. Oxidative stress intensifies the inflammatory response by activating inflammation-related signalling pathways and increasing the levels of pro-inflammatory cytokines [1,31]. Both inflammatory factors and oxidative stress are strongly associated with depression and SD [32,33]. In our study, PN was found to potentially alleviate brain damage caused by oxidative stress and improve cognitive function in SD mice by enhancing the activities of GSH, SOD, and CAT in the hippocampus. The PN also reduced serum levels of inflammatory factors in SD mice, confirming the results of our network pharmacology analysis [34,35]. Previous research has reported that piperine inhibits inflammation and oxidative stress through autophagy activation. Furthermore, cold-pressed oil extracted from PN has been identified as a potential antioxidant and neuroprotective agent, supporting our findings [16,36]. SD-induced inflammation causes damage to hippocampal neurons [37]. Damage to hippocampal neurons may lead to dysfunction of the neurotransmitter system, further exacerbating cognitive and emotional problems. In Nissl and HE staining, we reported that PN could reduce hippocampal damage. Piperine has been reported to exert neuroprotective effects in combination with quercetin as a bioenhancer, improving neurotransmitter levels for GABA, 5-HT, DA, and Glu [38]. GABA, a key regulator of sleep and immune responses, is found in high concentrations in the brain. It plays a vital role in maintaining the balance between excitation and inhibition within the nervous system, thereby supporting memory function and emotional stability [10,39]. Dysregulation of 5-HT and DA is commonly associated with psychiatric disorders such as anxiety and depression [40]. In addition to affecting cognitive function, SD often affects mood, so whether 5-HT and DA also play a role is a question worth exploring [11,41]. Glu is involved in the process of neural plasticity and is closely associated with the formation of learning and memory. Excessive release of glutamate can trigger overexcitation of neurons, leading to neuronal damage [42]. In our study, the levels of GABA, 5-HT, and DA decreased in the hippocampus of SD mice, while the level of Glu decreased significantly. Through the regulation of the neurotransmitter levels mentioned above, PN was able to mitigate damage in the mouse hippocampus. The JAK1/STAT3 pathway is an important pathway in cellular signal transduction that is widely involved in immune response, cell proliferation, differentiation, and functional regulation of the nervous system [43,44]. The excessive expression of inflammatory factors can activate the JAK1/STAT3 pathway, which may lead to hippocampal neuronal damage, impair learning and memory abilities, and exacerbate mood disorders [45]. In our study, PN may play an important role in SD by inhibiting the over-activation of the JAK1/STAT3 signalling pathway. The Jun gene encodes the c-Jun protein, which regulates gene expression in neural cells along with other stress-related proteins such as c-Fos [46]. Caspase3 plays a critical role in apoptosis. SD may trigger apoptosis by activating the caspase3 pathway, leading to neuronal damage and followed by a decline in cognitive function [47]. In vivo animal experiments and in vitro cellular studies further supported these findings, indicating that PN can improve learning and memory skills and neuronal damage by preventing inflammatory reactions and death.

## 4. Materials and Methods

### 4.1. Materials

Agomelatine (Les Laboratoires Servier Industrie, Gidy, France, HJ20150581), piperine (Shanghai Source Leaf Biological Technology Co., Ltd. (Shanghai, China)), tumour necrosis factor-alpha (TNF-α), interleukin-6 (IL-6), interleukin-1 beta (IL-1β), and cell counting kit-8 kits were provided by Solaibao Technology Co., Ltd. (Beijing, China). Superoxide dismutase (SOD), glutathione (GSH), catalase (CAT), and malondialdehyde (MDA) kits were provided by Nanjing Jiancheng Bioengineering Institute (Nanjing, China). The Hoechst 33,258 kit was provided by Beyotime Biotechnology Co., Ltd. (Shanghai, China). The TRUEscript RT MasterMix reverse transcription kit was provided by Ailide Biotechnology Co., Ltd. (Beijing, China). NovoStart SYBR qPCR SuperMix was provided by Nearshore Protein Technology Co., Ltd. (Suzhou, China). Janus kinase 1 (Jak1) and phosphorylated Jak1 (p-Jak1) antibodies were purchased from Cell Signaling Technology (Danvers, MA, USA). Signal transducer and activator of transcription 3 (STAT3), c-Jun proto-oncogene (c-Jun), Cysteinyl aspartate specific protease 3 (Caspase-3), and β-actin were purchased from Abcam (Cambridge, UK). PC12 cells (rat pheochromocytoma cells, high differentiation) were acquired from the BeNa culture collection (Beijing, China).

### 4.2. Extraction and Preparation of PN

Samples were obtained from the Hainan Academy of Agricultural Science and identified as PN fruits by Prof. Zhouwei Duan. After grinding the PN fruits and passing them through a 12-mesh sieve, the material was soaked overnight in 80% ethanol at a 1:12 (g/mL) ratio. The mixture was heated and refluxed three times, each for 1 h. After filtration, the filtrate was concentrated, and a thick paste was acquired, referred to as the PN.

### 4.3. Determination of Piperine in PN Using HPLC

The separation was performed on a Kromasil C18 (250 mm × 4.6 mm, 5 µm) column with methanol–water (74:26) as the mobile phase at a flow rate of 1.0 mL/min and a detection wavelength of 343 nm. The column temperature was maintained at room temperature.

### 4.4. Component Analysis of PN Using LC-MS/MS

Samples were analysed using an HSS-T3 column (2.1 × 100 mm, 1.8 µm) (Waters, Milford, MA, USA). In the positive ionisation mode, the aqueous phase consisted of water (A), while the organic phase (B) comprised acetonitrile. Both phases contained 0.1% formic acid. The aqueous phase of the negative ion mode consisted of water with 5 mM ammonium formate (A), while the organic phase was acetonitrile (B). The gradient elution protocol followed these steps: 0–1 min, 10% B; 1–9 min, 10–90% B; 9–10 min, 95% B; 10–11 min, 95–10% B; and 11–12 min, 10% B. The injection volume was 2 μL, the column’s temperature was 35 °C, and the flow rate was 0.3 mL/min. Mass spectrometry conditions are referred to in previous reports [48].

### 4.5. Network Pharmacology Analysis of PN

#### 4.5.1. Collection of Target Compounds from PN

Compounds identified by LC-MS/MS were queried through PubChem, and SwissADME was used to screen human-absorbable chemical components to identify active compounds. Subsequently, SwissTargetPrediction was used to forecast the possible biological targets of these active substances. The database ID is shown in Supplementary Table S1.

#### 4.5.2. Collection of Disease Targets

Using the keywords “sleep deprivation” and “depression”, gene expression profiles related to SD and depression were searched in the GEO database. GSE208668 and GSE98793 were selected as the study samples. The limma R package was employed to create volcano plots for data visualisation. After identifying potential genes and targets using the keywords “sleep deprivation” and “depression” across the OMIM, Drug Bank, DisGeNET, Gene Cards, and CTD databases, gene expression data from GEO were used for its validation. The most relevant candidate genes and targets were identified by intersecting the data from the aforementioned databases.

#### 4.5.3. Constructing the PPI Network

Cytoscape 3.8.2 was used to visualise the intersecting target data and perform network topology analysis with the CytoNCA plugin. Modular analysis was conducted using the MCODE plugin, while the cytoHubba plugin was used to calculate key targets, further identifying core heterogeneous genes and potential functional modules.

#### 4.5.4. GO and KEGG Enrichment Analyses

The intersecting targets (214) were subjected to GO and KEGG enrichment analyses and core target visualisation using R packages 3.4.0. Cytoscape 3.8.2 was used to build a target-pathway network.

#### 4.5.5. Molecular Docking Validation

The 3D structure of the ligand was obtained from the PubChem database. Molecular optimisation was performed using SYBYL-X 2.0 software. Receptor 3D structures were retrieved from the RCSB database. Receptors were pre-processed using mgltools_win32_1.5.6 software. The docking affinity between the receptor and ligand was tested using AutoDock Vina 1.1.2 software. Pymol 2.5.0 software was also used to visualise the results.

### 4.6. Establishment of an SD Mouse Model

ICR male mice (25 g, seven weeks) were purchased from Beijing Viton Lihua Laboratory Animal Company (Beijing, China), Licence No. SCXK (Beijing) 2021-0006. The breeding conditions were as described by previous researchers [49]. After five days of acclimatisation feeding, ICR mice were randomly divided into five groups: Control, Model, PN: 75 mg/kg, 150 mg/kg, and 300 mg/kg. Except for the standard group, the remaining groups of mice were placed in an automatic sleep interruption apparatus (SIA) to deprive the mice of sleep. The experimental methods were adapted from previous reports [50]. In brief, after three days of acclimatisation in the SIA (rotational speed of 1 revolution/min, with a 2 min rest after each revolution), modelling was performed on the model and experimental groups of mice. In contrast, the control group was reared under normal conditions. During the modelling period, mice could freely access food and water in the SIA and were administered medication once daily by gavage. After four weeks of modelling, corresponding behavioural tests were performed. The mice were immediately returned to the SIA after finishing the behavioural tests on the same day until all behavioural tests were completed. This work was authorised by the Animal Experiment Ethics Committee of the Institute of Food Science and Technology, Chinese Academy of Agricultural Sciences (SLXD-2023011728), and was conducted by the 3R principles and international guidelines for animal ethics.

### 4.7. Behaviour Tests

#### 4.7.1. Depressive Behaviour Test

The forced swim test (FST), tail suspension test (TST), and open field test (OFT) were employed to assess depression-like behaviours in SD mice. In the FST, the mice were submerged in a 30 cm deep, transparent cylindrical tank. After a 2 min acclimation period, an automated system recorded the total immobility time of 4 min [51]. In the TST, the mice were held aloft by their tails using sticky tape attached to a hook approximately 5 cm above the base. After a 2 min acclimatisation period, the instrument automatically measured the accumulated immobility time for a 4 min interval. From our previous experience, slight modifications were made, and the OFT was employed to assess the exploratory behaviour of SD mice [52]. The mice were placed facing the wall in the edge zone of a black circular arena (90 cm × 50 cm). At the same time, a real-time detection and analysis system recorded their movement for a specified duration.

#### 4.7.2. Cognitive Function Tests

Using the Morris water maze test (MWM), the cognitive abilities of SD mice were assessed [53]. The first phase, the escape acquisition phase, included the MWM test, consisting of our quadrants, with an invisible platform in one of the fixed quadrants located approximately 1.50 cm below the water surface. During a continuous five-day training session, the mice were positioned in the other three quadrants in succession, and their latency to locate the platform was recorded. After the completion of the escape acquisition phase, the second phase, the spatial exploration phase, was conducted. The platform was removed, and the animals were placed in water from the second quadrant. The number of entries was recorded in the original platform quadrant and other relevant metrics in 90 s.

### 4.8. Detection of Inflammatory Factors in SD Mice

Following the instructions provided by the kit, the serum levels of IL-6, IL-1β, and TNF-α were measured using the ELISA method. In short, antigens or antibodies were immobilised on a microplate, and the enzyme-labelled secondary antibody reacted with the substrate to generate a measurable colour change, allowing for the assessment of inflammation levels via absorbance.

### 4.9. Detection of Oxidative Markers in SD Mice

Mouse serum levels of MDA, SOD, CAT, and GSH were measured using the kit. In short, the steps were followed in the instruction manual to process the sample and evaluate the oxidation level by measuring its absorbance.

### 4.10. HE and Nissl Staining

Brain tissues were fixed in paraformaldehyde (4%). After fixation, the brain tissue underwent paraffin embedding and was sliced into coronal surfaces that were 5 µm thick. Subsequently, HE and Nissl staining was performed according to standard operating procedures.

### 4.11. Detection of Neurotransmitters in SD Mice

Neurotransmitters in the hippocampus were measured based on previous studies [54]. To conduct chromatographic separation, an Eclipse Plus C18 column (4.6 × 200 mm, 5 μm) was used at 35 °C. The mobile phase consisted of acetonitrile and formic acid water with a 0.2 mL/min flow rate. Multiple reaction monitoring was conducted in positive ionisation mode for the detection of each analyte, with *m*/*z* transitions as follows: GABA (104.0→69.0), 5-HT (176.9→136.02), Glu (148.0→84.0), and DA (154.0→137.0).

### 4.12. In Vitro Cell Model

Standard cell culture conditions were used, as previously mentioned [55]. In short, PC12 cells were cultured and kept in an incubator at 37 °C (5% CO_2_, 95% humidity) with RPMI-1640 medium, and the culture medium was transformed every two days. The corticosterone (CORT) was used to induce PC12 cells to establish an in vitro model. According to the kit instructions, the optimal induced concentration of corticosterone and the non-toxic concentration of PN were determined by measuring cell activity. Cell apoptosis was determined using the Hoechst 33,258 kit.

### 4.13. RT-qPCR for Detecting Core Gene Expression Levels

Total RNA was extracted from the hippocampus of mice according to the kit instructions, followed by cDNA synthesis using the TRUEscript RT MasterMix reverse transcription kit. Finally, RT-qPCR was performed using NovoStart SYBR qPCR SuperMix (Novoprotein, Suzhou, China) on a QuantStudio 5 RT-PCR detection system (Thermo fisher scientific, Waltham, MA, USA). The calculation was performed using the 2^−ΔΔCt^ method. Primer sequences are recorded in Supplementary Table S2.

### 4.14. Western Blot

Based on previous studies, proteins were extracted from hippocampal tissue, and their concentrations were determined using the BCA assay [56]. Proteins in equal quantities were separated on an SDS-polyacrylamide gel and then put onto a PVDF membrane. The membrane was blocked with 5% skim milk for 2 h. Primary antibodies, including Jak1 (3332, CST), p-Jak1 (3331, CST), STAT3 (ab68153, Abcam), c-Jun (ab40766, Abcam), Caspase-3 (ab184787, Abcam), and β-Actin (ab8226, Abcam), were included and left at 4 °C for the entire night. Following three TBST washes, the membrane was left to incubate for 2 h at room temperature with the secondary antibody. Protein signals were then detected using the ECL chemiluminescence method.

### 4.15. Data Analysis

GraphPad Prism 9.0 was used for statistical analysis, and the Tukey test and one-way analysis of variance were employed to evaluate intergroup differences. The mean values ± standard deviation (SD) were used to display the data. Statistical significance was deemed as a *p*-value < 0.05.

## 5. Conclusions

In summary, this study identified the main components of PN using LC-MS/MS, followed by predictive analysis of these components and their mechanisms of action through network pharmacology and molecular docking techniques. The results indicated that piperine, dibutyl phthalate, and pentaporphyrinl in PN might be the main active components related to the improvement of SD-induced memory impairment and depressive mood. STAT3, Jun, and Caspase3 are most likely primary targets of its action. Experimental validation results demonstrated that PN improves depressive mood and cognitive impairment in SD mice by inhibiting the over-activation of the JAK1/STAT3 pathway, reducing oxidative stress and inflammatory responses, and modulating neurotransmitters. Our study provides experimental support for the development of PN in functional foods and nutritional supplements, particularly highlighting its potential for antioxidation, anti-inflammation, and the enhancement of neurocognitive functions. However, this study has several limitations, including a focus on only three main compounds, the omission of potential sex differences in SD, and insufficient investigation into the ADME (absorption, distribution, metabolism, and excretion) of PN in plasma and brain tissues. Future research should broaden its scope to comprehensively explore the therapeutic potential of PN.

## Figures and Tables

**Table 1:** Major components in PN.

No.	Name	M/Z	RT/min	Pos/Neg
1	Piperine	286.1359	608	pos
2	Betaine	116.9269	667.3	neg
3	5-Hydroxyconiferaldehyde	177.0547	348	pos
4	Linoleic acid	280.2629	553.3	pos
5	9,10-Epoxyoctadecenoic acid	295.2279	448.5	neg
6	13-L-Hydroperoxylinoleic acid	293.2122	465.2	neg
7	Trans-1,2-Cyclohexanediol	115.9194	666.1	neg
8	(−)-alpha-Curcumene	203.1792	402.2	pos
9	(6Z)-Octadecenoic acid	281.2488	634.8	neg
10	4-Hydroxy-3-methoxy-benzaldehyde	151.0395	261.6	neg
11	D-Fructose	179.0572	412	neg
12	Fructose-1P	161.0426	534.9	neg
13	Caproic acid	114.9336	573.7	neg
14	All-trans-Retinoic acid	299.259	510.4	neg
15	Homogentisate	167.0343	117.8	neg

M/Z: mass-to-charge ratio; RT: retention time; pos: positive ion mode; neg: negative ion mode.

## Data Availability

All data and models generated or used during the study appear in the submitted article.

## Supplementary Materials

The following supporting information can be downloaded at: https://www.mdpi.com/article/10.3390/ijms26051842/s1.
