# Supplementary material for: The Extract of Piper nigrum Improves the Cognitive Impairment and Mood in Sleep-Deprived Mice Through the JAK1/STAT3 Signalling Pathway
Source: Int J Mol Sci. 2025 Feb 21;26(5):1842. doi: 10.3390/ijms26051842 (PMC11899911; doi:10.3390/ijms26051842)
Supplement: Supplementary file 1 [file ijms-26-01842-s001.zip › ijms-3419588-supplementary.pdf]

1、Database links table (Table S1)

2、Primer sequences used for RT-qPCR (Table S2)

3、Chemical Fingerprinting of PN extract (Figure. S1)

Table S1 Database links table

Databases	Web site
PubChem	https://pubchem.ncbi.nlm.nih.gov/
SwissTargetPrediction	http://www.swiss targetprediction.ch/index.php
GEO	http://www.ncbi.nlm.nih. gov/geo/
String database	https://cn.string-db.org/
AutoDock Vina 1.1.2 software	http://vina.scripps.edu/
RCSB	http://www.rcsb.org/

Table S2 Primer sequences used for RT-qPCR

Gene	Primer sequence	
MMP9	Forward	CGTCATTCGCGTGGATAAGG
	Reverse	TTTGGAAACTCACACGCCAG
STAT3	Forward	TACCAGCCCTCCAATCAAAG
	Reverse	GGTCACACAGCACACAATCC
JUN	Forward	CACCACTICCCCAACAGAT
	Reverse	TICCTCATGCGCTICCICICT
Caspase3	Forward	CTCGCTCTGGTACGGATGTG

	Reverse	TCCCATAAATGACCCCTTCATCA
PTGS2	Forward	CTGCGCCTTTTCAAGGATGG
	Reverse	GGGGATACACCTCTCCACCA
ALB	Forward	GATGAAACATATGIGTCCCCAAGA
	Reverse	TGTGTTCCTTAGGGIGTIGATITT
GAPDH	Forward	AGGTCGGTGTGAACGGATTTG
	Reverse	GGGGTCGTTGATGGCAACA

Figure. S1

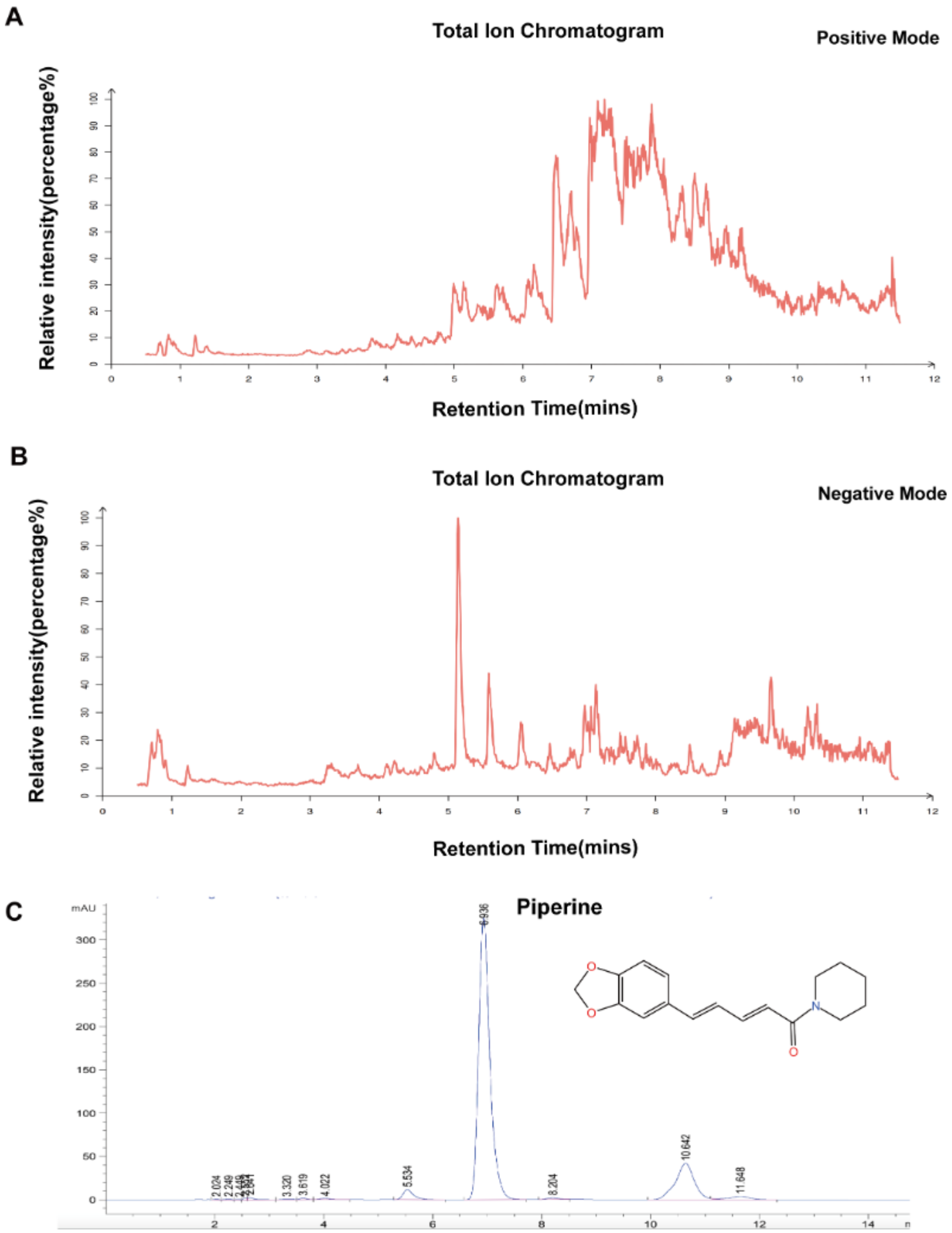

Figure. S1. Chemical Fingerprinting of PN extract. (A) Positive ion mode total ion chromatogram; (B) Negative ion mode total ion chromatogram; (C) Piperine content determination.
